# 2-Hy­droxy-3-meth­oxy­benzaldehyde (*o*-vanillin) revisited

**DOI:** 10.1107/S1600536812029571

**Published:** 2012-07-04

**Authors:** David Shin, Peter Müller

**Affiliations:** a Phillips Academy, 180 Main St, Andover, MA 01810, USA; b X-Ray Diffraction Facility, MIT Department of Chemistry, 77 Massachusetts Avenue, Building 2, Room 325, Cambridge, MA 02139-4307, USA

## Abstract

The structure of *ortho*-vanillin, C_8_H_8_O_3_, has been revisited with modern methods and at low temperature (100 K). The previous structure [Iwasaki *et al.* (1976 ▶). *Acta Cryst*. B**32**, 1264–1266] is confirmed, but geometric precision is improved by an order of magnitude. The C atom of the meth­oxy group lies close to the benzene ring plane, which is the most common geometry for –OMe groups lying *ortho* to –OH groups on an aromatic ring. The crystal structure displays one intra­molecular O—H⋯O and three weak inter­molecular C—H⋯O hydrogen bonds.

## Related literature

For the original structure of *o*-vanillin, see: Iwasaki *et al.* (1976 ▶). For C—H⋯acceptor inter­actions, see: Steiner (1996 ▶). For a summary of general refinement techniques applied, see: Müller (2009 ▶). For a description of the Cambridge Structural Database, see: Allen (2002 ▶).
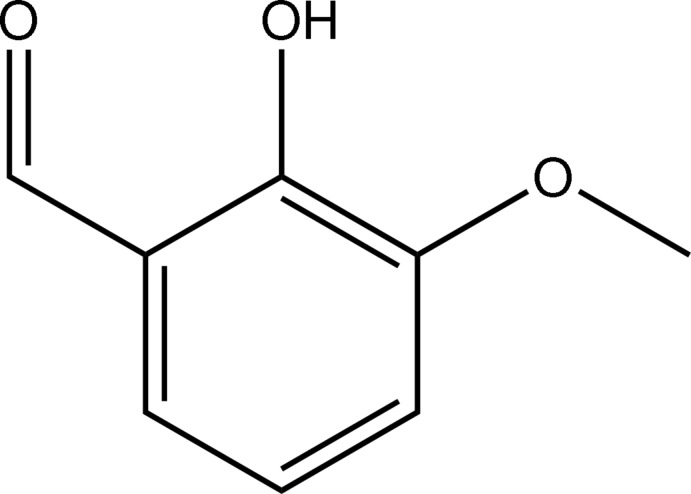


## Experimental

### 

#### Crystal data

C_8_H_8_O_3_
*M* *_r_* = 152.14
Orthorhombic, 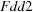

*a* = 24.367 (5) Å
*b* = 24.407 (3) Å
*c* = 4.7786 (5) Å
*V* = 2842.0 (7) Å^3^
*Z* = 16
Mo *K*α radiation
μ = 0.11 mm^−1^
*T* = 100 K
0.45 × 0.20 × 0.06 mm

#### Data collection

Bruker SMART APEXII CCD diffractometer
Absorption correction: multi-scan (*SADABS*; Sheldrick, 2009 ▶) *T*_min_ = 0.709, *T*_max_ = 1.000
21987 measured reflections
1203 independent reflections
1153 reflections with *I* > 2σ(*I*)
*R*_int_ = 0.053

#### Refinement

*R*[*F* ^2^ > 2σ(*F* ^2^)] = 0.034
*wR*(*F* ^2^) = 0.095
*S* = 1.11
1203 reflections
104 parameters
2 restraints
H atoms treated by a mixture of independent and constrained refinement
Δρ_max_ = 0.32 e Å^−3^
Δρ_min_ = −0.21 e Å^−3^

### 

Data collection: *APEX2* (Bruker, 2011 ▶); cell refinement: *SAINT* (Bruker, 2011 ▶); data reduction: *SAINT*; program(s) used to solve structure: *SHELXS97* (Sheldrick, 2008 ▶); program(s) used to refine structure: *SHELXL97* (Sheldrick, 2008 ▶); molecular graphics: *SHELXTL* (Sheldrick, 2008 ▶); software used to prepare material for publication: *SHELXTL*and *PLATON* (Spek, 2009) ▶.

## Supplementary Material

Crystal structure: contains datablock(s) I, global. DOI: 10.1107/S1600536812029571/hb6851sup1.cif

Structure factors: contains datablock(s) I. DOI: 10.1107/S1600536812029571/hb6851Isup2.hkl

Supplementary material file. DOI: 10.1107/S1600536812029571/hb6851Isup3.cdx

Supplementary material file. DOI: 10.1107/S1600536812029571/hb6851Isup4.cml

Additional supplementary materials:  crystallographic information; 3D view; checkCIF report

## Figures and Tables

**Table 1:** Selected torsion angles (°)

C8—O3—C3—C4	7.5 (2)
C8—O3—C3—C2	−172.98 (13)

**Table 2:** Hydrogen-bond geometry (Å, °)

*D*—H⋯*A*	*D*—H	H⋯*A*	*D*⋯*A*	*D*—H⋯*A*
O2—H2⋯O1	0.85 (2)	1.84 (2)	2.6017 (17)	148 (2)
C8—H8*B*⋯O1^i^	0.98	2.56	3.064 (2)	112
C6—H6⋯O3^ii^	0.95	2.53	3.374 (2)	149
C7—H7⋯O3^ii^	0.95	2.59	3.414 (2)	146

Symmetry codes: (i) 
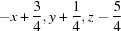
; (ii) 
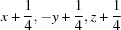
.

**Table 3:** Comparison (Å) between the room and low temperature structures of *o*-vanillin

parameter	room tempreature	low temperature (this study)
*R*1[*I*>2*σ*(*I*)]	0.077	0.034
C1—C7	1.427	1.452 (2)
O1—C7	1.229	1.233 (2)
O2—C2	1.354	1.3535 (17)
O3—C3	1.364	1.3689 (16)
O3—C8	1.430	1.434 (2)

**Fig. 1.**
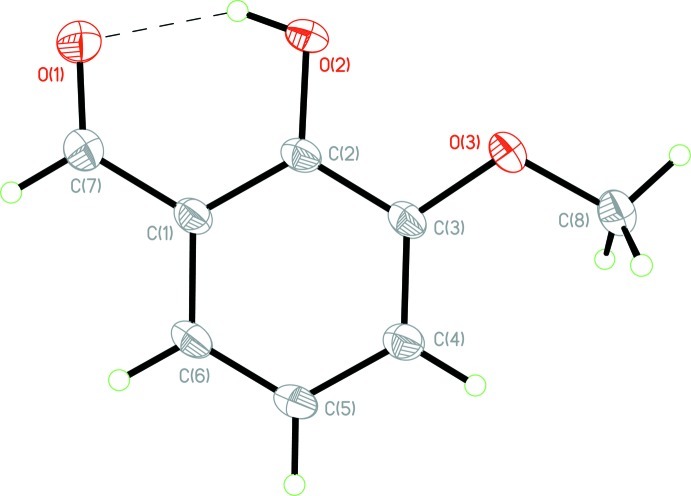
The molecular structure of the title compound with displacement ellipsoids shown at the 50% probability level.

**Fig. 2.**
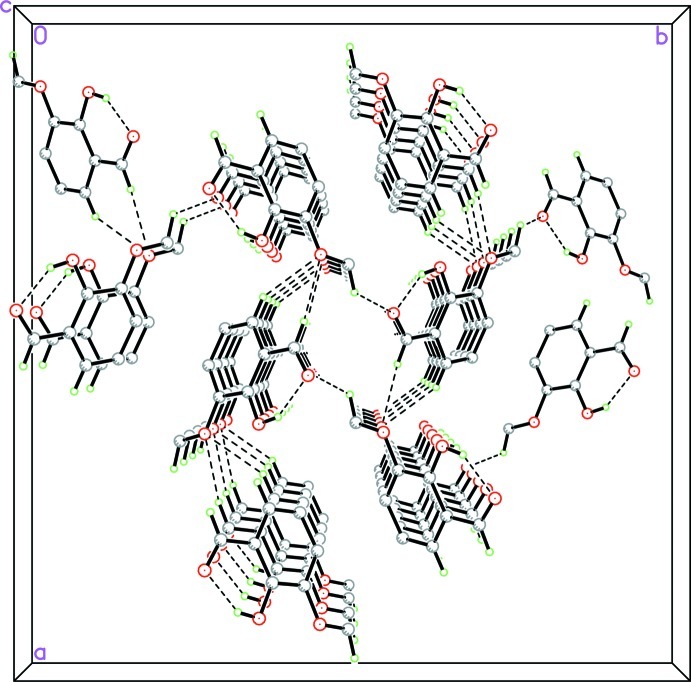
Packing diagram of the title compound in a projection along the c axis. Hydrogen bonds are shown as dashed lines. This projection was chosen for easier comparison with Figure 3 in Iwasaki et al. (1976).

**Fig. 3.**
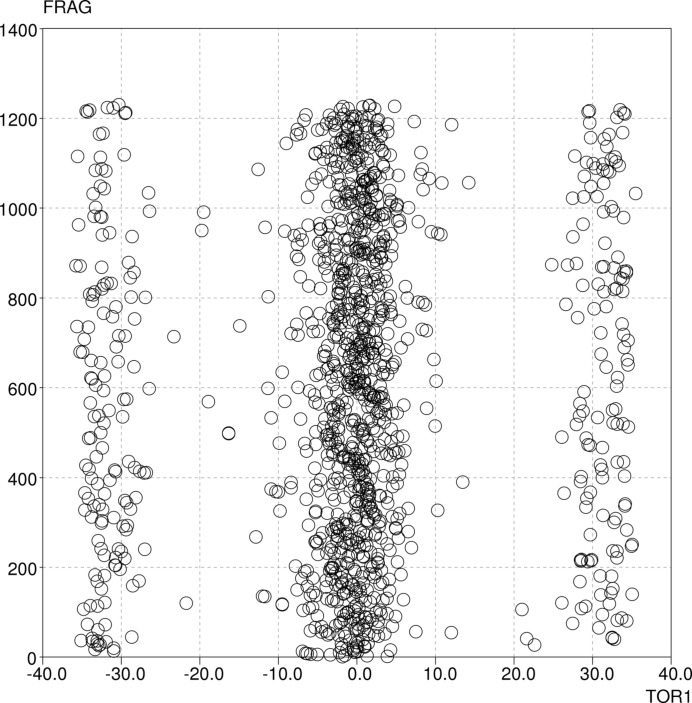
Scatterplot showing ranges of torsion angles on ortho substituted methoxy-hydroxy aryls. 74.4% of the torsion angles are close to zero (as in the structure of o-vanillin), while 22.4% are reported to be close to ±30°.

## supplementary crystallographic information

### Comment

The only published crystal structure of *o*-vanillin was determined almost 40 years ago at room temperature and based on Weissenberg films (Iwasaki *et al.* 1976). This structure has been redetermined to current standards with modern methods and state-of-the-art equipment. No discrepancies were found betweeen the two structures, however the residual values of the refinement as well as standard uncertainties on bond lengths and angles are significantly improved in the modern structure. Iwasaki *et al.* (1976) report standard uncertainties for bonds between non-hydrogen atoms of 0.011 Å to 0.03 Å, while standard uncertainties in this study range from 0.0016 Å to 0.002 Å. The residual value of the refinement for "observed reflexions" in the structure by Iwasaki *et al.* (1976) is reported as 0.077, while the low-temperature structure could be refined to an *R*1 of 0.034 for *I*>2*σ(I)* (see also Table 3).

The atomic labeling scheme for non-hydrogen atoms was chosen to match that of Iwasaki *et al.* (1976) and a direct comparison between the structures reveals that most bond distances were found to be somewhat shorter in the room temperature structure than at low temperature (see Table 3). This is probably a result of libration effects, which are more pronounced at higher temperatures. Iwasaki *et al.* (1976) report the bond between C5 and C6 to be significantly shorter than all other bonds in the benzene ring and point out that this was a phenomenon observed in other related structures (namely iso-vanillin and 2,6-dinitrophenol). Indeed, the bond between C5 and C6 is the shortest of the six aromatic bonds, however in the low-temperature structure there are four long and two short aromatic C—C bonds. The long bonds range from 1.403 (2) to 1.409 (2) Å, the short ones are 1.385 (2)Å (C3—C4) and 1.379 (2) Å (C5—C6).

The benzene ring of *o*-vanillin is almost planar in the solid state, with a root-mean-square deviation of 0.0054Å. The methoxy group is rotated out of this plane by about 7° (see Table 1). This is a common torsion angle for methoxy groups standing *ortho* to hydroxyl functions. A search of the Cambridge Structure Data Base (Allen, 2002) for this geometric feature (only error free, organic structures with *R*-values below 10% were considered) resulted in 1230 hits (from 825 structures) and the torsion angles in question cluster around two different values: 0° and ± 30°. Figure 3 shows a scatterplot of all results from this search. 915 of the 1230 hits (74.4%) fall between -10° and +10°, with an average torsion angle of -0.163° while 276 (22.4%) belong to the ±30° torsion angle group (average values calculated separately for the ranges -25° to -35° and 25° to 35° are -31.586° and 31.397°, respectively), and 39 hits (3.2% of all hits) fall outside the specified ranges.

In addition to the obvious intramolecular hydrogen bond between O1 and O2 that was already described by Iwasaki *et al.* (1976), the program *PLATON* (Spek, 2009) finds three weak C—H···O interactions (Steiner, 1996, Table 2). One of them, C8—H8B···O1^i^, connects the *o*-vanillin molecules into almost flat, indefinite zigzag chains extending parallel to the *b*-*c* plane. The other two, C6—H6···O3^ii^ and C7—H7···O3^ii^, crosslink those chains, giving rise to the supramolecular network shown in Figure 2 (symmetry codes: i: 3/4 - *x*, 1/4 + *y*, -5/4 + *z*; ii: 1/4 + *x*, 1/4 - *y*, 1/4 + *z*).

### Experimental

Ortho-vanillin was obtained from Fluka in the form of yellow plates and used without recrystallization.

### Refinement

The structure was refined against *F*^2^ on all data by full-matrix least squares with *SHELXL97* (Sheldrick, 2008), following established refinement strategies (Müller, 2009). All non-hydrogen atoms were refined anisotropically. All hydrogen atoms binding to carbon were included into the model at geometrically calculated positions (C—H target distance 0.98 Å for methyl hydrogen atoms, 0.95 Å for all others) and refined using a riding model. The torsion angle of the methyl group was allowed to refine. Coordinates for the hydrogen atom on O2 were taken from the difference Fourier synthesis. This hydrogen atom was subsequently refined semi-freely using a distance resraint for the O—H bond (target value 0.84 (2) Å). The *U*_iso_ values of all hydrogen atoms were constrained to 1.2*U*_eq_ (1.5 times for hydroxyl and methyl H atoms) of the respective atom to which the hydrogen atom bonds. Anomalous dispersion was negligible and Friedel pairs were merged before refinement.

### Crystal data

**Table:**

C_8_H_8_O_3_	*D*_x_ = 1.422 Mg m^−^^3^
*M**_r_* = 152.14	Mo *K*α radiation, λ = 0.71073 Å
Orthorhombic, *F**d**d*2	Cell parameters from 9845 reflections
*a* = 24.367 (5) Å	θ = 2.4–30.5°
*b* = 24.407 (3) Å	µ = 0.11 mm^−^^1^
*c* = 4.7786 (5) Å	*T* = 100 K
*V* = 2842.0 (7) Å^3^	Plate, yellow
*Z* = 16	0.45 × 0.20 × 0.06 mm
*F*(000) = 1280	

### Data collection

**Table:**

Bruker SMART APEXII CCD diffractometer	1203 independent reflections
Radiation source: IµS micro-focus sealed tube	1153 reflections with *I* > 2σ(*I*)
Incoatech IµS multilayer optics monochromator	*R*_int_ = 0.053
Detector resolution: 8.3 pixels mm^-1^	θ_max_ = 30.5°, θ_min_ = 2.4°
φ and ω scans	*h* = −34→34
Absorption correction: multi-scan (*SADABS*; Sheldrick, 2009)	*k* = −34→34
*T*_min_ = 0.709, *T*_max_ = 1.000	*l* = −6→6
21987 measured reflections	

### Refinement

**Table:**

Refinement on *F*^2^	Primary atom site location: structure-invariant direct methods
Least-squares matrix: full	Secondary atom site location: difference Fourier map
*R*[*F*^2^ > 2σ(*F*^2^)] = 0.034	Hydrogen site location: inferred from neighbouring sites
*wR*(*F*^2^) = 0.095	H atoms treated by a mixture of independent and constrained refinement
*S* = 1.11	*w* = 1/[σ^2^(*F*_o_^2^) + (0.0544*P*)^2^ + 2.2598*P*] where *P* = (*F*_o_^2^ + 2*F*_c_^2^)/3
1203 reflections	(Δ/σ)_max_ < 0.001
104 parameters	Δρ_max_ = 0.32 e Å^−^^3^
2 restraints	Δρ_min_ = −0.21 e Å^−^^3^

### Special details

**Table:**

Experimental. Low-temperature diffraction data were collected on a Bruker-AXS X8 Kappa Duo four-circle diffractometer coupled to a Smart Apex2 CCD detector, performing φ and ω scans, using Mo K_α_ radiation (λ=0.71073 Å) from an Incoateac IµS micro-source. The instrument was purchased with the help of funding from the National Science Foundation (NSF) under Grant Number CHE-0946721.
Geometry. All e.s.d.'s (except the e.s.d. in the dihedral angle between two l.s. planes) are estimated using the full covariance matrix. The cell e.s.d.'s are taken into account individually in the estimation of e.s.d.'s in distances, angles and torsion angles; correlations between e.s.d.'s in cell parameters are only used when they are defined by crystal symmetry. An approximate (isotropic) treatment of cell e.s.d.'s is used for estimating e.s.d.'s involving l.s. planes.
Refinement. Refinement of *F*^2^ against ALL reflections. The weighted *R*-factor *wR* and goodness of fit *S* are based on *F*^2^, conventional *R*-factors *R* are based on *F*, with *F* set to zero for negative *F*^2^. The threshold expression of *F*^2^ > σ(*F*^2^) is used only for calculating *R*-factors(gt) *etc*. and is not relevant to the choice of reflections for refinement. *R*-factors based on *F*^2^ are statistically about twice as large as those based on *F*, and *R*- factors based on ALL data will be even larger.

### Fractional atomic coordinates and isotropic or equivalent isotropic displacement parameters (Å^2^)

**Table:**

	*x*	*y*	*z*	*U*_iso_*/*U*_eq_	
O1	0.45674 (5)	0.05966 (5)	1.0153 (3)	0.0291 (3)	
O2	0.38924 (4)	0.12815 (5)	0.7797 (3)	0.0247 (2)	
H2	0.3995 (10)	0.1013 (8)	0.880 (5)	0.037*	
O3	0.37296 (4)	0.20682 (5)	0.4128 (3)	0.0245 (3)	
C1	0.48543 (6)	0.12138 (6)	0.6601 (4)	0.0202 (3)	
C2	0.43287 (5)	0.14455 (6)	0.6257 (3)	0.0195 (3)	
C3	0.42513 (5)	0.18616 (6)	0.4251 (3)	0.0199 (3)	
C4	0.46886 (6)	0.20316 (6)	0.2616 (3)	0.0213 (3)	
H4	0.4635	0.2311	0.1258	0.026*	
C5	0.52111 (6)	0.17949 (6)	0.2945 (4)	0.0226 (3)	
H5	0.5507	0.1910	0.1795	0.027*	
C6	0.52928 (6)	0.13949 (6)	0.4940 (3)	0.0225 (3)	
H6	0.5648	0.1241	0.5191	0.027*	
C7	0.49383 (6)	0.07907 (6)	0.8706 (4)	0.0248 (3)	
H7	0.5300	0.0656	0.8980	0.030*	
C8	0.36098 (6)	0.24468 (7)	0.1915 (4)	0.0273 (3)	
H8A	0.3821	0.2784	0.2194	0.041*	
H8B	0.3217	0.2533	0.1925	0.041*	
H8C	0.3709	0.2282	0.0114	0.041*	

### Atomic displacement parameters (Å^2^)

**Table:**

	*U*^11^	*U*^22^	*U*^33^	*U*^12^	*U*^13^	*U*^23^
O1	0.0248 (5)	0.0320 (5)	0.0306 (6)	−0.0029 (4)	0.0006 (5)	0.0070 (5)
O2	0.0143 (4)	0.0318 (5)	0.0280 (6)	−0.0018 (4)	0.0044 (5)	0.0051 (5)
O3	0.0145 (4)	0.0297 (5)	0.0293 (6)	0.0034 (4)	−0.0005 (4)	0.0041 (5)
C1	0.0142 (5)	0.0247 (6)	0.0218 (6)	−0.0006 (4)	−0.0003 (5)	−0.0001 (6)
C2	0.0135 (5)	0.0237 (6)	0.0213 (6)	−0.0025 (4)	0.0009 (5)	−0.0007 (5)
C3	0.0127 (5)	0.0249 (6)	0.0222 (6)	0.0002 (4)	−0.0009 (5)	−0.0010 (5)
C4	0.0173 (6)	0.0244 (6)	0.0222 (7)	−0.0020 (5)	0.0006 (5)	0.0001 (6)
C5	0.0150 (5)	0.0281 (6)	0.0248 (7)	−0.0020 (5)	0.0036 (6)	−0.0007 (6)
C6	0.0131 (5)	0.0284 (6)	0.0261 (8)	−0.0006 (4)	0.0004 (5)	−0.0013 (6)
C7	0.0210 (6)	0.0273 (6)	0.0261 (7)	−0.0001 (5)	−0.0017 (6)	0.0021 (6)
C8	0.0212 (6)	0.0305 (7)	0.0304 (8)	0.0058 (5)	−0.0016 (6)	0.0029 (7)

### Geometric parameters (Å, º)

**Table:**

O1—C7	1.233 (2)	C4—C5	1.4070 (19)
O2—C2	1.3535 (17)	C4—H4	0.9500
O2—H2	0.850 (17)	C5—C6	1.379 (2)
O3—C3	1.3689 (16)	C5—H5	0.9500
O3—C8	1.434 (2)	C6—H6	0.9500
C1—C6	1.403 (2)	C7—H7	0.9500
C1—C2	1.4094 (19)	C8—H8A	0.9800
C1—C7	1.456 (2)	C8—H8B	0.9800
C2—C3	1.409 (2)	C8—H8C	0.9800
C3—C4	1.385 (2)		
			
C2—O2—H2	107.6 (18)	C6—C5—H5	120.0
C3—O3—C8	117.24 (13)	C4—C5—H5	120.0
C6—C1—C2	119.99 (14)	C5—C6—C1	120.28 (13)
C6—C1—C7	120.49 (13)	C5—C6—H6	119.9
C2—C1—C7	119.52 (14)	C1—C6—H6	119.9
O2—C2—C3	118.53 (12)	O1—C7—C1	123.86 (14)
O2—C2—C1	122.13 (14)	O1—C7—H7	118.1
C3—C2—C1	119.35 (13)	C1—C7—H7	118.1
O3—C3—C4	125.45 (14)	O3—C8—H8A	109.5
O3—C3—C2	114.77 (12)	O3—C8—H8B	109.5
C4—C3—C2	119.78 (12)	H8A—C8—H8B	109.5
C3—C4—C5	120.67 (14)	O3—C8—H8C	109.5
C3—C4—H4	119.7	H8A—C8—H8C	109.5
C5—C4—H4	119.7	H8B—C8—H8C	109.5
C6—C5—C4	119.92 (14)		
			
C2—C1—C7—O1	3.8 (3)	C8—O3—C3—C2	−172.98 (13)
C8—O3—C3—C4	7.5 (2)	C6—C1—C7—O1	−176.85 (16)

### Hydrogen-bond geometry (Å, º)

**Table:**

*D*—H···*A*	*D*—H	H···*A*	*D*···*A*	*D*—H···*A*
O2—H2···O1	0.85 (2)	1.84 (2)	2.6017 (17)	148 (2)
C8—H8*B*···O1^i^	0.98	2.56	3.064 (2)	112
C6—H6···O3^ii^	0.95	2.53	3.374 (2)	149
C7—H7···O3^ii^	0.95	2.59	3.414 (2)	146

Symmetry codes: (i) −*x*+3/4, *y*+1/4, *z*−5/4; (ii) *x*+1/4, −*y*+1/4, *z*+1/4.

### Comparison (Å) between the room and low temperature structures of *o*-vanillin.

**Table:**

parameter	room tempreature	low temperature (this study)
*R*1[*I*>2*σ*(*I*)]	0.077	0.034
C1—C7	1.427	1.452 (2)
O1—C7	1.229	1.233 (2)
O2—C2	1.354	1.3535 (17)
O3—C3	1.364	1.3689 (16)
O3—C8	1.430	1.434 (2)

## References

[bb1] Allen, F. H. (2002). *Acta Cryst.* B**58**, 380–388. 10.1107/s0108768102003890 12037359

[bb2] Bruker (2011). *APEX2* and *SAINT* Bruker AXS Inc., Madison, Wisconsin, USA.

[bb3] Iwasaki, F., Tanaka, I. & Aihara, A. (1976). *Acta Cryst.* B**32**, 1264–1266.

[bb4] Müller, P. (2009). *Crystallogr. Rev.* **15**, 57–83.

[bb5] Sheldrick, G. M. (2008). *Acta Cryst.* A**64**, 112–122. 10.1107/S0108767307043930 18156677

[bb6] Sheldrick, G. M. (2009). *SADABS* University of Göttingen, Germany.

[bb7] Spek, A. L. (2009). *Acta Cryst.* D**65**, 148–155. 10.1107/S090744490804362X PMC2631630 19171970

[bb8] Steiner, Th. (1996). *Crystallogr. Rev.* **6**, 1–57.

